# Neuroprotection as a Therapeutic Target for Diabetic Retinopathy

**DOI:** 10.1155/2016/9508541

**Published:** 2016-03-31

**Authors:** Cristina Hernández, Massimo Dal Monte, Rafael Simó, Giovanni Casini

**Affiliations:** ^1^CIBERDEM (CIBER de Diabetes y Enfermedades Metabolicas Asociadas) and Diabetes and Metabolism Research Unit, Vall d'Hebron Institut de Recerca (VHIR), Universitat Autonoma de Barcelona, Passeig Vall d'Hebron 119-129, 08035 Barcelona, Spain; ^2^Department of Biology, University of Pisa, Via San Zeno 31, 56127 Pisa, Italy; ^3^Interdepartmental Research Center Nutrafood “Nutraceuticals and Food for Health”, University of Pisa, Via del Borghetto 80, 56124 Pisa, Italy

## Abstract

Diabetic retinopathy (DR) is a multifactorial progressive disease of the retina and a leading cause of vision loss. DR has long been regarded as a vascular disorder, although neuronal death and visual impairment appear before vascular lesions, suggesting an important role played by neurodegeneration in DR and the appropriateness of neuroprotective strategies. Upregulation of vascular endothelial growth factor (VEGF), the main target of current therapies, is likely to be one of the first responses to retinal hyperglycemic stress and VEGF may represent an important survival factor in early phases of DR. Of central importance for clinical trials is the detection of retinal neurodegeneration in the clinical setting, and spectral domain optical coherence tomography seems the most indicated technique. Many substances have been tested in animal studies for their neuroprotective properties and for possible use in humans. Perhaps, the most intriguing perspective is the use of endogenous neuroprotective substances or nutraceuticals. Together, the data point to the central role of neurodegeneration in the pathogenesis of DR and indicate neuroprotection as an effective strategy for treating this disease. However, clinical trials to determine not only the effectiveness and safety but also the compliance of a noninvasive route of drug administration are needed.

## 1. Introduction

Diabetic retinopathy (DR) is a multifactorial progressive disease of the retina with high social impact and with an extremely complex pathogenesis that involves a variety of different cells, molecules, and factors. Metabolic changes in the diabetic retina result in altered expression pattern of a number of mediators including growth factors, neurotrophic factors, cytokines/chemokines, vasoactive agents, and inflammatory and adhesion molecules, resulting in vascular lesions and cell death [1–4]. In the recent past, DR was generally considered a purely vascular disorder of the retina and visual impairment was attributed to vascular damage. In the last few years it has become evident that considerable damage of retinal neurons is also present in early stages of DR. As the investigations proceed and data are produced, it appears that neurodegeneration also plays a significant role in microvascular impairment [5–9].

The present review focuses on the possible causes and the effects of neuronal damage in DR and discusses possible approaches based on neuroprotection for the treatment of this disease. In particular, with this review of the literature we invite the reader to consider the following hypotheses: (i) neuronal damage directly induced by diabetic stress may be responsible for functional abnormalities in visual function; (ii) a central role in linking neuronal damage to early microvascular damage (i.e., vascular leakage) may be played by VEGF expressed and released by damaged neurons; (iii) therapeutic strategies based on neuroprotection will be useful in preventing or arresting DR development.

## 2. Neurodegeneration in DR and the Role of VEGF

### 2.1. Evidence of Retinal Neuronal Damage in DR

There is evidence that death of neurons appears before that of vascular cells in the retina of both humans with DR and experimental animal models of DR [10], while recent data in db/db diabetic mice reported ganglion cell apoptosis, retinal thinning, and ERG deficits in the absence of obvious microvascular changes [11]. These observations support the view that neuronal damage is an early event in the pathogenesis of DR [12, 13] and that DR may be considered a neurodegenerative eye disease [14]. Only in a few cases the occurrence of extensive neuronal apoptosis in DR has not been fully acknowledged [15, 16] and the only report excluding any loss of retinal ganglion cells in DR is based on an analysis of cell numbers in the GCL of retinal sections [17], which may have been unfit to detect significant changes. Actually, the vast majority of papers document the presence of apoptotic markers and/or the appearance of functional defects in diabetic retinas. In particular, apoptotic markers, including caspase-3 [18, 19], caspase-9 [19], Bax [18, 19], Bad [20], and Fas [18], have been observed to increase in retinal ganglion cells of patients with diabetes, while enhanced release of cytochrome c and expression of apoptosis inducing factor has been documented both in ganglion cells and in photoreceptor cells [20]. Consistent with these findings, optical coherence tomography (OCT) observations in patients with type 1 or type 2 diabetes revealed reduced thickness of the inner retinal layers, which was only minimally associated with vascular lesions [21–23]. Interestingly, a recent work, based on a retrospective analysis of spectral domain OCT (SD-OCT) scans of a number of subjects with diabetes and with various stages of DR, shows that thinning of the inner retina appears early in the pathology and before any visible vascular signs of DR can be appreciated [24], suggesting the opportunity of neuroprotective interventions to prevent chronic neurodegeneration. Experimental data in animal models of DR indicate that uncontrolled insulin-deficient diabetes markedly increases neuronal cell death in the retina [10, 25, 26]. For instance, in rats with streptozotocin-induced diabetes, a significant increase in caspase-3 expression has been reported in the nerve fiber layer, ganglion cell layer, and inner plexiform layer as early as after two weeks from diabetes induction [27]. An increase in the cytosol concentration of cytochrome c, secondary to the activation of caspase system, has also been observed in streptozotocin-exposed rats after 8 months from treatment [28]. In addition, a significant reduction of total retinal thickness and a decreased number of ganglion cells together with increased active caspase-3 and TUNEL labeling were reported in rat and mouse models of type 2 diabetes [11, 29, 30]. Suffering of retinal neurons in diabetic rodent retinas is also suggested by altered expression of proteins involved in axonal transport in retinal neurons [31] and by the decrease in the content of synaptic proteins in retinal nerve terminals [32–34].

The neural cell types suffering alterations in early DR have been studied in more detail in different rodent models of DR. A number of investigations reported increased apoptosis of retinal ganglion cells [10, 26, 35–38]. Cholinergic [39], dopaminergic [37, 39, 40], and neuronal nitric oxide synthase-expressing [41] amacrine cells have been also observed to be affected in DR. Interestingly, a recent study reported significant reduction of dopamine levels in rodent models of DR, while visual function was improved by treatments with l-DOPA or with dopaminergic receptor agonists [42]. In addition, alterations in retinal glutamatergic and GABAergic systems are suggested by the observed early transient changes in the content of glutamate and GABA vesicular transporters in retinal synaptosomes [43]. Other data documented photoreceptor death [37, 44, 45] and alterations in the expression of phototransduction proteins [46], while a reduction in photoreceptor density close to the fovea has also been reported by a recent study in adult humans with diabetes [47]. However, the data relative to photoreceptor death or damage in diabetic retinas are not conclusive. Indeed, a number of investigations in animal models of DR reported lack of photoreceptor death and clinical studies failed to convincingly demonstrate photoreceptor damage in diabetic patients, as summarized in a recent review [48]. The presence of discrepancies about possible photoreceptor damage in DR may be explained, at least in some cases, assuming that observed photoreceptor loss or damage is due to factors other than diabetes (for instance, strain differences, in animal studies) or that the effects on photoreceptors are critically dependent on the duration of diabetes.

In addition to changes in the cell bodies (e.g., TUNEL-positive nuclei and caspase-3 activation, indicating apoptosis), defects in the processes of retinal neurons are also evident in the retinas of rodent models of DR, and they are represented mainly by abnormal swellings observed in ganglion cell axons [49, 50] and morphologic changes in the dendrites of ON-type ganglion cells [50]. Observed signs of damage also include ganglion cell body swelling and axonal fragmentation [50] and closely mimic the pathologic features observed in postmortem human retinas from patients with diabetes [51]. Impairments at the level of ganglion cell axons are also documented by the finding of reduced efficiency of both retrograde and anterograde axonal transports and of reactive gliosis of axonal astrocytes in the optic nerve of diabetic rats [52, 53].

The damage to retinal neurons in early phases of DR is likely to be responsible for the functional deficits observed both in animal models of DR and in patients with diabetes. For instance, in diabetic db/db mice, retinal thinning and ganglion cell apoptosis have been found to correlate with pattern ERG alterations [11]. This assumption is also supported by the observations reported in a recent retrospective case-control cohort study including eyes of diabetic patients with resolved diabetic macular edema (DME) and eyes of age-matched diabetic patients without maculopathy. Using measures of retinal thickness, obtained with SD-OCT, and measures of visual acuity, this study showed a significant correlation between thinning of the ganglion cell layer and visual acuity in patients with resolved DME, suggesting that retinal alterations occurring in DME and DR may lead to permanent visual deficiency [54]. Studies in animal models showed that, in rats with experimentally induced diabetes, ganglion cells exhibit the greatest level of dysfunction within the components of the electroretinogram (ERG) [55], while significant decreases in the rod and cone ERG components have been also reported [45, 56]. ERG abnormalities have been reported in db/db diabetic mice [29], while ERG deficits have been recorded in streptozotocin treated rats as early as 2 weeks after diabetes induction [57], indicating that functional abnormalities may be due to malfunctioning of neurons starting well before the appearance of clear signs of apoptotic cell death. In diabetic patients, earlier studies reported abnormalities in the ERG [58] and, more recently, a number of electrophysiological investigations in patients with diabetes, but with no or mild signs of vascular damage, have identified deficits of retinal functions in multiple layers of the retina [59–63]. In addition, color vision and contrast sensitivity are also affected in patients with diabetes [64, 65]. Electrophysiological and psychophysical observations have recently reported abnormalities of retinal function in diabetic patients without any evidence of microvascular changes [66]. In adolescents and young adults with type 1 diabetes but without or with mild nonproliferative DR, as well as in type 1 diabetic patients with normal ocular fundus and absent breakdown of the blood-retina barrier, multifocal ERG (mfERG) showed global retinal dysfunction [67, 68], confirming that changes in retinal function occur before the onset of vascular pathology.

Together, these data document the important role that neurodegenerative phenomena may play in the retina of a diabetic subject before the classical signs of DR are recognizable and support the view that a neuroprotective strategy should be considered for the treatment of patients before the appearance of vascular lesions.

### 2.2. Possible Causes of Neurodegeneration in DR

A logic consequence of the observation that neurons are damaged in DR is the search for the possible causes of this fact. A knowledge of the mechanisms that are likely to affect neuronal survival in the diabetic retina is an essential prerequisite to design therapeutic strategies aimed at reducing the impact of diabetes on retinal neurons.

#### 2.2.1. Advanced Glycation End-Products (AGEs)

AGEs are late products of nonenzymatic glycation. The deposition of hyperglycemia-induced AGEs in retinal blood vessels plays an important role in the onset and development of DR. AGEs and their receptors (RAGEs) have been detected in virtually all cellular types of the diabetic retina [69]. The AGE-RAGE axis plays a central role in the inflammation and microvascular dysfunction in DR [70], since AGEs have been found to stimulate apoptosis of retinal pericytes [71, 72] and of vascular endothelial cells [73, 74]. However, the potential effects of these compounds on the survival of retinal neurons are not fully elucidated. RAGE activation may induce retinal neuron apoptosis through activation of nitric oxide synthase [75] or may activate astrocytes and Müller glia promoting the expression of proinflammatory cytokines [76] that would contribute to neuronal death. A correlation between AGE and neuronal death is suggested by the observations that treatments reducing AGE formation also result in significant reduction of neuronal death in retinas of db/db diabetic mice [77] and that in diabetic rats AGE blockade prevents both ERG abnormalities and ganglion cell apoptosis [78]. A direct evidence of detrimental effects of AGEs on retinal neurons is provided by results showing that AGEs induce neuronal death in nondiabetic retinal explants of both rat [79, 80] and mouse [81] retinas.

#### 2.2.2. Glutamate Excitotoxicity

Glutamate is the major excitatory neurotransmitter in the retina and glutamate excitotoxicity is the process damaging or killing neurons expressing NMDA or AMPA receptors due to excessive receptor stimulation and calcium influx secondary to altered levels of glutamate in the extracellular space. Elevated glutamate levels have been observed in experimental models of diabetes [82–84], where alterations of the expression of glutamate receptors and calcium binding proteins have also been reported [85–88]. An increase in glutamate levels has been detected in the vitreous of patients with advanced DR [89, 90], while an immunohistochemical analysis in human retinas of donor eyes from subjects with diabetes described changes in the expression of glutamate receptor subunits [91]. Together, these data indicate that diabetes disrupts retinal glutamate homeostasis and that in DR excitotoxic phenomena may cause serious damage to retinal neurons. The reasons for extracellular accumulation of glutamate in DR may involve different mechanisms. For instance, Müller cells may become less efficient in performing glutamate uptake [92] or in converting glutamate to glutamine due to reduced levels of the enzyme glutamine synthetase [82, 83], while a decreased oxidation of glutamate to alpha-ketoglutarate has also been reported in diabetic retinas [84].

#### 2.2.3. Intracellular Pathways

The polyol pathway is activated in the presence of elevated intracellular glucose levels and it is linked with the progression of DR [93]. The rate-limiting enzyme in this pathway is aldose reductase, which produces sorbitol from glucose causing reduction of NADPH levels and depletion of glutathione. This glutathione depletion and the consequent increased oxidative stress are major inductors of retinal damage. The polyol pathway also promotes other mechanisms of cellular damage, including AGE formation and activation of the protein kinase C (PKC) pathway, which may lead to inflammation and growth factor imbalances [94]. Thus the polyol pathway can be regarded as a mediator of glucose toxicity that is responsible for a wide spectrum of abnormalities, including neural, glial, and vascular damage, detectable during the development of DR [95]. In line with this view, diabetic mice deficient in aldose reductase were protected from diabetes-induced impairments in contrast sensitivity and spatial frequency threshold [96]. In addition, the cellular membranes are impermeable to sorbitol and the inability of sorbitol to efflux out of the cell leads to its intracellular accumulation, causing cellular damage mainly due to osmotic imbalance. Although the exact mechanism of sorbitol-induced cell death is still uncertain, this process is likely to promote the progression of DR [97].

Poly(ADP ribose) protein (PARP), a nuclear enzyme involved in the regulation of a variety of cellular events, is another potential inductor of retinal damage in DR. Indeed, PARP is activated in diabetic retinas causing DNA damage and oxidative stress [98–100], while its inhibition results in increased neurotrophic support and reduction of neuronal damage in early experimental DR [101].

Hyperglycemia and diabetes also increase the function of the hexosamine biosynthetic pathway, which results in many changes in both gene and protein levels, contributing to the pathogenesis of DR [102]. In particular, the increased glucose flux through the hexosamine pathway may cause apoptosis of retinal neurons in two ways, that is, via induction of apoptosis due to altered glycosylation of proteins or via impairment of the neuroprotective effect of insulin mediated by Akt [103].

Activation of PKC is known to be implicated in several pathologic changes occurring in DR, such as basement membrane thickening, extracellular matrix expansion, vascular permeability, apoptosis, angiogenesis, leukocyte adhesion, and cytokine activation [2]. Similar to activation of the hexosamine pathway, also PKC activation is likely to result in retinal cell damage due to inhibition of insulin-induced Akt activation [104].

#### 2.2.4. Oxidative Stress

A considerable increase of oxidative stress is known to occur in the diabetic retina [105, 106] and it is the likely cause of neurodegeneration [107]. The metabolic pathways discussed above are associated with mitochondrial overproduction of reactive oxygen species (ROS); therefore, oxidative stress may be regarded as a sort of final common pathway for the glucose-induced abnormalities found in DR. Oxidative stress may also result from nitrative stress [108, 109], activation of polyol or hexosamine pathways, or uncoupling of endothelial nitric oxide synthase [110]. In addition, oxidative stress and AGE production appear to be interrelated mechanism, since AGEs can induce oxidative stress through direct or indirect stimulation of ROS generation, while AGE synthesis is accelerated by oxidative stress [105, 111]. Similarly, relationships between oxidative stress and glutamate release have also been reported [84]. In summary, oxidative stress is likely to increase the flux of polyol pathway as well as AGE and RAGE expression, to induce PKC overactivation and endothelial cell apoptosis and to promote inflammation [105]. Enhanced ROS levels may also cause retinal neuronal damage by reducing the expression of brain derived neurotrophic factor (BDNF). Indeed, in retinas of mice with streptozotocin-induced diabetes, reductions of both BDNF and synaptophysin have been reported, but these reductions were attenuated by the antioxidant lutein [107].

#### 2.2.5. The Retinal Renin-Angiotensin System (RAS)

The most well-known function of RAS is regulation of blood pressure as well as of fluid and electrolyte content in the body. The classical RAS is a systemic hormonal system, but local RASs have been identified in a number of organs, including the eye and the retina [112]. In DR, RAS is upregulated, with increased levels of renin, angiotensin converting enzyme (ACE), and angiotensin receptors (ATR) [113]. Retinal RAS is likely to be implicated in the development of vascular lesions in DR. Indeed, inhibitors of ACE, ATR, or aldosterone synthase reduce retinal neovascularization and VEGF expression in rodent models of DR and some benefit of RAS blockade in DR has been reported in clinical trials [114]. In retinas of diabetic animals, ACE inhibition and/or AT1R blockade have been reported to attenuate functional deficits [115–117] and to reduce oxidative stress, neurodegeneration, and mitochondrial dysfunction [118], indicating that RAS activation in DR may also affect retinal neurons.

#### 2.2.6. Neuroinflammation

A considerable body of evidence from animal models and patients shows that DR is a chronic low-grade inflammatory disorder with participation of inflammatory mediators [119]. Dysregulation of metabolic pathways resulting in excessive mitochondrial ROS production, increased oxidative stress, and RAS activation leads to upregulation of a variety of growth factors and inflammatory cytokines, chemokines, prostaglandins, and inflammatory cells such as macrophages and neutrophils in a complex chain of events. The resulting alterations decrease vascular wall integrity, increasing vascular permeability, lumen occlusion, and ischemia [119, 120]. Neuroinflammation involves the activation of microglia and astroglia. In contrast to microglia activation in acute inflammation, which may have beneficial effects, in chronic neuroinflammation microglia activation, resulting in release of proinflammatory mediators and increased oxidative stress [121], contributes to the pathogenesis of neurodegenerative diseases [122]. While the retinal microglia is likely to play important roles in DR, there is also strong evidence that Müller cells are major sources of inflammatory mediators [123] and become activated in response to a variety of pathological changes in the retina [124]. In particular, an analysis of gene expression in Müller cells from retinas of diabetic rats revealed 78 altered genes, of which one-third were associated with inflammation [125]. Sustained neuroinflammation creates a toxic milieu that may lead to detrimental effects in neuronal cells [126, 127], although the mechanisms by which these cytokines may contribute to neural apoptosis in DR have not been fully clarified.

#### 2.2.7. Endoplasmic Reticulum (ER) Stress

ER stress results from an impairment of the folding capacity of the ER causing accumulation of unfolded proteins in the ER lumen and activation of the unfolded protein response (UPR). ER stress contributes to increased oxidative stress as well as inflammation and apoptosis may occur upon failure of UPR to resolve the ER stress. This condition is likely to be involved in the pathogenesis of different neuronal diseases in brain and retina, including the early stages of DR [128]. Disruption of glutamate homeostasis with consequent glutamatergic overstimulation, which has been described in DR (see above), may activate ER stress and apoptosis in retinal ganglion cells of mouse retinas [129]. In addition, data obtained using streptozotocin-treated rats reported that the expression of ER stress marker proteins in the retina is significantly increased by diabetes and that reducing ER stress results in protection of diabetic retinas from neuronal cell death and vascular damage [130].

#### 2.2.8. Dysregulation of Endogenous Neuroprotectants

Insulin provides trophic support for retinal neurons via phosphatidylinositide 3-kinase/Akt and P70S6 kinase pathways [131–133]. This constitutive insulin prosurvival signaling in the retina is impaired by diabetes and may contribute to neuronal degeneration in DR [134]. Similarly, insulin-like growth factors (IGFs) are neurotrophic factors that may be involved in the pathogenesis of DR. Indeed IGF-1 mRNA has been found to be reduced in the eye in early stages of clinical and experimental diabetes [135], while IGF replacement therapy counteracts proapoptotic abnormalities preceding retinal cell degeneration in diabetic rats [136].

Pigment epithelium-derived factor (PEDF) has been described as a multifunctional protein with neuroprotective, antiangiogenic, antivasopermeability, anti-inflammatory, and antioxidative effects [137]. Its levels are decreased in aqueous or vitreous of patients with diabetes, suggesting that a decrease of retinal PEDF levels in humans may contribute to the development of DR [138]. Consistent with the hypothesis that a reduction of PEDF may contribute to retinal neuronal death in DR,* in vitro* studies in retinal Müller cells suggest that PEDF could increase glutamine synthetase expression and prevent downregulation of glutamate transporter in these cells, thereby limiting glutamate excitotoxicity in the diabetic retina [139, 140].

Alterations in retinal levels of neurotrophins (NTs) and their receptors (tropomyosin receptor kinases, Trks, and p75 neurotrophin receptor, p75^NTR^) may be present in DR. Indeed, in the retina of streptozotocin-treated rats, the upregulation of NT-3 and NT-4 and of their receptors TrkA and TrkB is associated with the progression of DR [141]. In addition, impaired maturation of the nerve growth factor (NGF) precursor proNGF, caused by the oxidative milieu of the diabetic retina, results in increased expression of proNGF, with a corresponding decrease in NGF. The dysregulation of proNGF maturation in the diabetic retina may contribute to diabetes-induced retinal ganglion cell death through the reduction in trophic support due to decreased NGF expression or through the direct activation of proapoptotic pathways in ganglion cells by proNGF interaction with the p75^NTR^ receptor [38, 142, 143]. Another pathway by which proNGF may cause ganglion cell death involves paracrine effects of proNGF/p75^NTR^-mediated secretion of tumor necrosis factor-*α* by Müller cells [144]. Reduction of retinal levels of BDNF has also been reported in animal models of diabetes [40, 101, 107, 145], probably caused, at least in part, by oxidative stress [107]. This reduction may affect retinal neuronal survival, since exogenous supply of BDNF has been found to reduce neurodegeneration in an animal model of DR [40].

Interphotoreceptor retinoid-binding protein (IRBP) is a photoreceptor-secreted glycolipoprotein that is essential for photoreceptor survival [146]. IRBP levels are reduced in the vitreous, while both IRBP mRNA and protein are significantly lower in the retinas of donors with diabetes than in those of nondiabetic donors [147, 148]. In addition, IRBP levels have been found to be negatively correlated with the levels of neurodegeneration in the retinas of patients with diabetes [148].

The neuropeptide somatostatin (SST), together with its receptors, is expressed in the retina, where it is involved in a variety of functions [149]. A downregulation of SST retinal expression has been reported in DR and it has been found to be associated with retinal neurodegeneration [150]. A decreased expression of SST in the retina results in marked decrease of intravitreal SST in the eyes of patients with proliferative DR [151, 152] or with diabetic macular edema [153]. SST is likely to play a dual action in DR, inhibiting both neoangiogenesis and neuronal death [149]. In particular, a major cause of neuronal death in diabetic retinas is increased extracellular glutamate and excitotoxicity, and SST may limit the extent of neuronal damage through inhibition of glutamate release [154–157] and of glutamate transporter downregulation [157].

### 2.3. VEGF: A Proangiogenic or a Neuroprotective Factor?

**Figure 1:**
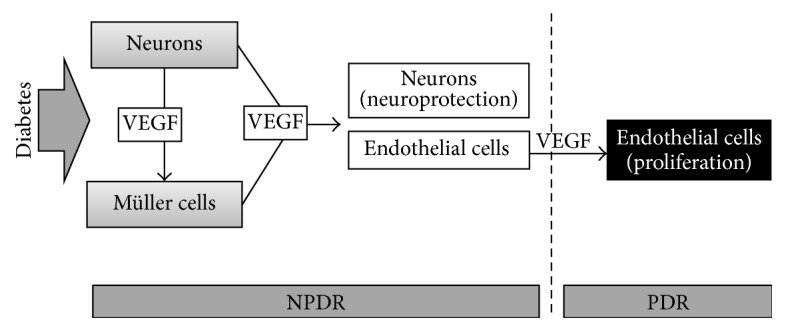
Possible role of VEGF in early DR. Under diabetic stress, VEGF would be immediately released by neurons and by Müller cells, which perhaps receive stimulation by the VEGF of neuronal origin. In the nonproliferative phase of DR (NPDR), released VEGF would act on retinal neurons as a neuroprotectant, while it would also bind to its receptors on endothelial cells. A prolonged interaction of VEGF with endothelial cells would lead to the proliferative phase of DR (PDR).

Observations of the brain after ischemia suggest that brain damage, in addition to the activation of death pathways, also stimulates protective mechanisms to counteract the expansion of the injury. Although the damaging effectors in the end prevail, the evidence suggests that concomitant self-protective mechanisms are elicited, implying that the central nervous system possesses intrinsic cytoprotective mechanisms that are likely to be mediated by chemical signals derived from the injured brain itself [158]. VEGF may be one of such signals released by the retina in the early phases of DR. Indeed, we have shown that retinal neurons in* ex vivo* ischemic retinas not only undergo cell death but also express and release VEGF, which, in turn, is likely to bind to its receptors expressed by endothelial cells [156, 159]. These observations indicate that VEGF expression and release are among the earliest responses of suffering retinal neurons and that, in this context, the significance of VEGF release is unlikely to be related to its proangiogenic effects. Rather, VEGF expression and release are probably related to a protective strategy of the retina, which tries to protect its cells from damage. Consistent with this view, glutamate excitotoxicity, one of the major causes of retinal neuronal death in DR, has been reported to upregulate VEGF production in diabetic retinas [160], while inhibition of NMDA receptors results in a decrease in vitreoretinal VEGF in diabetic rats [161]. In addition, we have recently observed that VEGF expression and release markedly increase in retinal explants within a few days of incubation in the presence of high glucose, oxidative stress, or AGE and that these increases are abolished or significantly reduced by the addition of neuroprotectants [81]. Together, these observations suggest that, in an early phase of DR, VEGF is likely to be expressed and released to rescue and protect retinal neurons. In this phase, VEGF would not act as a proangiogenic but as a prosurvival factor. Interestingly, it has been reported that the optic nerve and retinal ganglion cells in a rat model of experimental glaucoma are protected from degeneration by short-term hyperglycemia [162]. Increased VEGF levels may negatively affect vessel permeability and the blood-retina barrier. Then, if the stressing conditions remain for a long time, the persistent high levels of VEGF may lead to vessel proliferation (Figure 1). In the retina, VEGF neutralization has been reported to cause apoptosis of retinal cells and loss of retinal function [163], while VEGF blockade has been observed to significantly increase neuronal cell death in a hypertensive glaucoma model [164]. Repeated intravitreal injections of bevacizumab have been found to cause extensive neuronal loss in the rat retina [165]. Furthermore, VEGF has been found to be able to rescue retinal neurons after optic nerve axotomy [166] and to play a role in the survival of photoreceptors and of Müller cells [163]. Finally, using organotypic retinal explants cultured in hypoxic conditions, we have recently demonstrated that decreased VEGF release is accompanied by increased retinal cell apoptosis, while increased VEGF release results in reduced rate of retinal cell death [167]. Therefore, the possibility exists that the VEGF released by the retina in acute stress conditions, as we have demonstrated in* ex vivo* ischemic models of the retina [156, 159], may have the scope of protecting retinal neurons rather than that of promoting angiogenesis. One may wonder why, despite high levels of VEGF expression and release in diabetic retinas, neuronal death is still present, resulting in functional abnormalities. The possibility exists that the diabetic milieu may impair VEGF function uncoupling its survival effect, as suggested by investigations with retinal endothelial cells under high glucose or hypoxia [168, 169]. In addition, a shift in VEGF splice variants reducing neuroprotective [170] and increasing proangiogenic VEGF isoforms [171], as well as the loss of other neuroprotective factors (i.e., somatostatin and IRBP) induced by diabetes, is other mechanisms promoting neuronal death even when an enhancement of VEGF does exist. VEGF neuroprotective actions in the retina are likely to be mediated by VEGF receptor 2 signaling via the phosphoinositide-3-kinase/Akt pathway, as demonstrated in different experimental models [164].

## 3. Therapeutic Perspectives for Neuroprotection in DR

### 3.1. Methods for Detecting Retinal Neurodegeneration in the Clinical Setting: A New Cornerstone for Clinical Trials?

Improvements in diabetes care and management have been crucial in lowering the incidence and severity of DR. One limiting factor that has hampered the investigation of new drugs for DR is the low sensitive endpoints in the clinical trials, which require large sample sizes as well as a long duration (about 5 years) to achieve a sufficient statistical power to detect potential treatment effectiveness. Best corrected visual acuity (BCVA) and derived variables are the only endpoints that have served as the basis for regulatory approval of retinal drugs. However, BCVA captures only a small portion of visual function and patients with good BCVA may have difficulties with daily activities such as reading or driving. Therefore, there is now widespread recognition of the need for improved endpoints for DR research.

Since neurodegeneration is an early event in the pathogenesis of DR that could participate in the development of microvascular impairment [14, 66], the study of the underlying mechanisms leading to neurodegeneration and the identification of mediators in the cross-talk between neurodegeneration and microangiopathy are essential for the development of new therapeutic strategies. Currently, the presence of neurodegeneration can be measured by SD-OCT, which allows the examination of morphological changes (i.e., the thinning of the ganglion cell layer or of the nerve fiber layer), or by means of functional methods such as mfERG, standard automated perimetry, frequency doubling perimetry, or microperimetry.

Among the methods for assessing the functional impairment, the mfERG is the gold standard. This is a noninvasive technique providing a topographic measure of retinal electrophysiological activity. The use of mfERG has provided compelling evidence suggesting a direct link between neural dysfunction and vascular abnormalities in DR. Thus, a delayed mfERG implicit time predicts the development of early microvascular abnormalities [59, 172]. In addition, it should be noted that the implicit time is spatially associated with microvascular abnormalities, correlates with retinopathy severity, and is a predictor for the development of visible vascular impairment over 1-year [173, 174] or 3-year period [172]. However, mfERG is a time-consuming and cumbersome examination and, consequently, its use is currently limited to pilot studies and clinical trials.

The current method for exploring the morphological changes in retina is the SD-OCT, which provides an anatomical image of the retina and complements the functional information obtained with mfERG. The reduction in the thickness of the ganglion cell layer and of the nerve fiber layer are the main parameters detected in DR and they have been observed even before any microvascular abnormality appears in the fundoscopic examination [66]. Hyperreflective intraretinal spots (HRS) can also be found in diabetic eyes without microangiopathy. HRS are mainly located in the inner retina, where the resident microglia are present and may represent a surrogate of microglial activation in the early stages of DR [175]. At present, SD-OCT seems to be the most practical method for monitoring neurodegeneration in DR.

In recent years a noninvasive instrument to assess mitochondrial function using flavoprotein autofluorescence has been developed. This method is based on the fact that the retina is particularly susceptible to oxidative stress because of high energy demands and light exposure. Before apoptosis, mitochondria exhibit impaired electron transport by energy generating enzymes in the respiratory chain, causing increased percentages of flavoproteins in the chain to be oxidized and rendered capable of absorbing blue light and emitting green autofluorescence [176]. Since oxidative stress and mitochondrial dysfunction are implicated in the pathogenesis of DR, it is reasonable to expect that flavoprotein autofluorescence may be used for monitoring retinal damage [177]. However, clinical studies to validate this method are needed.

### 3.2. New Therapeutic Strategies Based on Administration of Neurotrophic Factors

**Figure 2:**
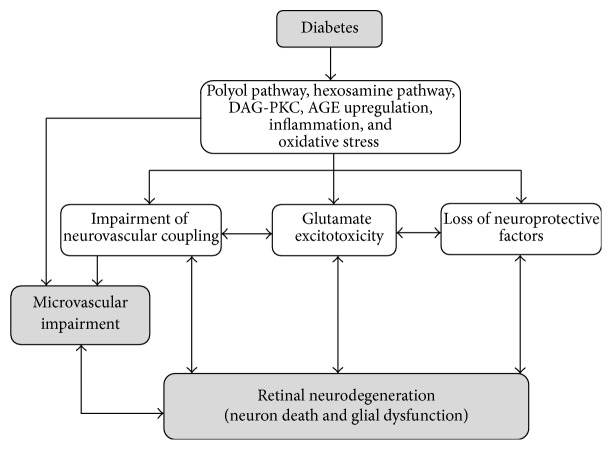
Potential therapeutic targets based on pathogenic mechanisms involved in retinal neurodegeneration induced by diabetes.

There are several therapeutic strategies based on the main pathogenic mechanisms involved in retinal neurodegeneration that, theoretically, could be implemented (Figure 2). However, systemically administered drugs blocking these pathways can hardly reach the retina at pharmacological concentrations and, in addition, could have serious adverse effects. On the other hand, when the early stages of DR are the therapeutic target, it would be inconceivable to recommend an aggressive treatment such as intravitreal injections. For all these reasons, topical treatment could be envisaged as a revolutionary treatment.

The use of eye drops has not been considered an appropriate route for the administration of drugs aimed at preventing or arresting DR because of the general assumption that in this way the drugs do not reach the posterior chamber of the eye (i.e., the vitreous and the retina). However, there is emerging evidence showing that several topically administered compounds are able to reach the retina in pharmacological concentrations, at least in animal models [178–181]. In addition, topical administration of drugs limits their action to the eye and minimizes associated systemic effects [182].

Adherence to ophthalmic treatments has a unique set of challenges compared to oral medications [183]. It must be noted that self-administering drops (i.e., for glaucoma treatment) requires coordination, manual dexterity, eye-hand coordination, and good vision. In addition, diabetic patients are commonly receiving a lot of treatments due to the high prevalence of comorbidities. However, it must be noted that a significant barrier to adherence is lack of information (failing to explain the benefit of a medication adequately). In this regard, it has been demonstrated that patients are more likely to be adherent to their medication if they understand the disease and the rationale for treatment. Therefore, educating the patient and his/her family could improve patient adherence to topical ocular therapy.

Pathologic conditions, including those characterizing the diabetic status, are known to affect retinal levels of neuroprotective agents, thus impairing the balance between neurotoxic and neuroprotective factors and resulting in damage to the neural retina. In this respect, many different substances have been tested for their neuroprotective properties in models of retinal stress similar to that in DR. Since a comprehensive review of all the substances employed in experimental studies is virtually impossible, we will briefly consider the potential use in DR of endogenous neuroprotective substances or of phytochemicals with antioxidant and anti-inflammatory properties.

#### 3.2.1. Endogenous Neuroprotective Substances

Insulin, IGF-1, PEDF, SST, pituitary adenylate cyclase activating peptide (PACAP), glucagon-like peptide- (GLP-) 1, and NTs are potential neuroprotective factors for the treatment of DR.

Subconjunctival insulin administration restores prosurvival signaling and reduces the rate of retinal cell death in streptozotocin-induced diabetic rats [184]. In this regard, it has been reported that insulin hydrogels can be implanted subconjunctivally for long term insulin delivery to the eye without any adverse events in rats [185]. IGFs are neurotrophic factors implicated in the pathogenesis of diabetic neurological disorders and treatment with IGF-1 analogs prevents early retinal biochemical abnormalities implicated in the progression of DR [136].

PEDF is a peptide with neurotrophic properties produced by retinal pigment epithelium cells and found to have neuroprotective effects towards photoreceptors [186]. In addition, systemic administration of PEDF to diabetic rats prevents Müller cell activation and retinal dysfunction [187]. On the other hand, PEDF is also a potent antiangiogenic factor [188] and intravitreal PEDF effectively reduces VEGF-induced vascular permeability in a mouse model of nonproliferative DR [189]. In addition, PEDF also exerts antioxidant and anti-inflammatory effects and reduces oxidative stress and the production of inflammatory markers in DR models [190]. Moreover,* in vitro* studies in retinal Müller cells suggest that PEDF decreases glutamate excitotoxicity induced by the diabetic milieu by increasing the expression of glutamine synthetase and by preventing glutamate transporter downregulation [139, 140]. Finally, intravitreal administration of PEDF upregulates glutamine synthetase and glutamate transporter expression and decreases glutamate levels in hypoxia [191]. Taken together, these findings suggest that multitarget molecules such as PEDF may be suitable candidates for new therapeutic approaches to treat DR. However, PEDF size may limit its utility as a topical therapeutic agent and, therefore, some synthetic PEDF-derived peptides containing biologically active fragments are needed. In this regard, the amino acid residues of PEDF contributing to the inhibition of VEGF-induced vascular permeability [189] and a PEDF fragment inhibiting retinal vascularization in an oxygen-induced retinopathy model [192] have been identified. Regarding DR, the topical administration (eye drops) of antiangiogenic PEDF60–77 and neuroprotective PEDF78–121 derivatives reduced neurodegeneration and microvascular leakage in the Ins2(Akita) mouse [193]. Furthermore, eye drop delivery of PEDF-34 promotes ganglion cell survival and axon regeneration after optic nerve crush injury in rats [194].

The neuropeptide SST and its five receptor subtypes (sst_1_–sst_5_) are expressed in the retina, where they are involved in multiple functions [195]. SST acting at sst_2_ expressed by bipolar cells may control glutamate release in the retina [154, 155, 196], suggesting that SST may protect neurons from apoptosis caused by extracellular glutamate levels in DR. In this respect, the first evidence has been provided that topical administration of SST prevents retinal neurodegeneration in streptozotocin-induced diabetic rats [157]. The main mechanism involved in this beneficial effect is indeed the reduction of glutamate-induced excitotoxicity. With this basis, a multicentric, phases II-III, randomized controlled clinical trial (EUROCONDOR) to assess the efficacy of SST administered topically (eye drops) to prevent or arrest retinal neurodegeneration is ongoing (EudraCT number: 2012-001200-38). This study has been funded by the European Commission in the setting of the FP7-HEALTH-2011 and the results will be available in April 2016.

PACAP is known to be protective against a variety of insults in mammalian retinas [197]. In particular, neuroprotective effects of PACAP against retinal cell loss induced by ischemia have been reported both* in vivo* and* in vitro* [156, 198]. In addition, neurodegeneration of several retinal cell types, including dopaminergic amacrine cells and ganglion cells, is counteracted by PACAP in streptozotocin-treated rats [37].

GLP-1 exerts neuroprotective effects in both central and peripheral nervous systems [199]. It has been shown that intravitreal injections of exendin-4 (a GLP-1R agonist) prevent ERG abnormalities and morphological features related to neurodegeneration in rats with streptozotocin-induced diabetes [200] and in Goto-Kakizaki rats [201]. Recently, abundant expression of GLP-1 receptor (GLP-1R) in human retinas has been observed [202]. In addition, both neuroprotection and prevention of vascular leakage have been reported using topical administration (eye drops) of GLP-1R agonists in the db/db mouse model [202].

Originally identified as trophic factors for neurons, NTs are a family of structurally and functionally related proteins that are essential for the growth, differentiation, and survival of several cell types including retinal neurons, glia, and endothelial cells [144]. The main members of this family are BDNF and NGF. In diabetic rats, retinal levels of BDNF are decreased, and intravitreal administration of exogenous BDNF rescues amacrine cells from neurodegeneration [40]. In addition, overexpression of BDNF in streptozotocin-induced diabetic rats, obtained by intraocular injection of an adenoassociated virus vector plasmid carrying the expression cassette of brain BDNF, resulted in enhanced ganglion cell survival and function [203]. The neuroprotective effect of BDNF in the retina seems to be mediated, at least in part, by prevention of the cytotoxic swelling of retinal glial and bipolar cells [204]. NGF plays an important role in neurodegeneration, inflammation, vascular permeability, and injury, all important processes in the pathogenesis of DR. There is no report on retinal production of NGF. However, its administration in rat models of diabetes is effective in counteracting retinal neurodegeneration. Indeed, intraocular injections of NGF prevent apoptosis in retinal ganglion cells and in Müller cells as well as pericyte loss and the formation of acellular capillaries [205]. Recently, applications of NGF as eye drops have been reported to protect retinal ganglion cells from degeneration in models of experimental glaucoma or DR [206].

#### 3.2.2. Antioxidant and Anti-Inflammatory Agents

Many papers suggest that oxidative stress plays a major role in the pathogenesis of DR. Therefore, the use of antioxidants may be viewed as potential therapeutics in the treatment of DR. Many phytochemicals have been extensively examined, although much more work is needed to definitely assess their effectiveness as alternative therapies in DR.

Flavonoids possess antioxidant, antiangiogenic, and anti-inflammatory properties; thus selected flavonoids may be effective in the prevention or treatment of ocular diseases, including DR [207]. In this respect, quercetin reduces oxidative stress, neuroinflammation, and apoptosis in streptozotocin-treated rats. Indeed, six-month treatment with quercetin restores glutathione levels and the activities of antioxidant enzymes, reduces the levels of inflammatory cytokines, and protects ganglion cells from apoptotic cell death [208]. Numerous studies have investigated the role of resveratrol in preventing or treating diabetic complications, including DR. Four-month oral administration of resveratrol to streptozotocin-treated rats partially prevents the decrease in antioxidant defenses, retinal thinning, production of inflammatory cytokines, and retinal cell apoptosis, without affecting plasma levels of insulin [209, 210]. Resveratrol also reduces retinal expression of genes involved in angiogenesis, inflammation, and oxidative stress in diabetic rats [211]. The antioxidant and antiangiogenic actions of curcumin have been regarded as suitable characteristics in order to propose curcumin as a novel drug for the treatment of DR. Two-month administration of curcumin to streptozotocin-treated rats inhibits the expression of VEGF [212], while three-month treatment with curcumin inhibits oxidative stress protects Müller cells and prevents the downregulation of glutamine synthase in the retina of diabetic rats [213]. A four-month treatment with curcumin induces significant hypoglycemic activity, reduces the decrease in glutathione levels and in the activity of antioxidant enzymes, decreases inflammatory factor levels, and prevents the structural degeneration and increase in capillary basement membrane thickness in the retina of diabetic rats [214]. Treatment with the flavonoid hesperetin for six months rescues the retina from oxidative stress, neuroinflammation, and apoptosis in diabetic rats [215]. Genistein, administered to streptozotocin-treated rats, attenuates retinal inflammation by targeting microglial activation [216] and confers protection against gliopathy and vasculopathy [217]. Interestingly, oral administration of genistein in association with *α*-lipoic acid and vitamins to preretinopathic diabetic patients, increases plasma levels of antioxidants, and ameliorates electroretinographic recordings [218]. Finally, epigallocatechin gallate protects the retina against glutamate toxicity through an antioxidant mechanism in diabetic spontaneously hypertensive rats [219].

Carotenoids are also powerful antioxidants. In particular, lutein and zeaxanthin, which in diabetes are decreased in serum and retina, have been reported to inhibit diabetes-induced retinal oxidative damage [220, 221]. Treatment with zeaxanthin has been tested in a large trial on age-related macular degeneration with moderate success [222].

Overall, data from literature indicate phytochemicals (not only flavonoids or carotenoids, as briefly described here, but also phenolic acids, terpenoids, etc.) as effective molecules in the management of retinal complications in diabetes, although further investigations using human clinical studies are needed to confirm the beneficial effects of phytochemicals in the treatment of DR.

Given that diabetic murine models do not develop advanced vascular abnormalities, the experimental studies mentioned above have demonstrated the usefulness of neuroprotective agents to prevent the development of retinal neurodegeneration and initial vascular leakage. Although it could be reasonable to speculate that neuroprotection of the retina will be useful at any stage of DR, further evidence demonstrating this issue is needed. In particular, more work both in animal models, using sufficiently large numbers of animals to have statistical power, and in the clinics is necessary to provide conclusive demonstration that neuroprotection not only preserves neurons from death or damage but also results in actual functional improvements.

### 3.3. Other Approaches

#### 3.3.1. Improving the Neurovascular Coupling Function

Alterations of the neurosensory retina and retinal vasculature are integrally linked, and the study of the interactions between blood vessels and the neurosensory retina is crucial to understand the pathogenesis of DR. Neurovascular coupling is the intrinsic physiological mechanism by which neural activity is coupled to blood flow and metabolism, thus enabling the retina to regulate blood flow in response to neural activity or metabolic demands. Indeed, the increased delivery of oxygen in response to the increased activity of retinal neurons is a consequence of the coupling between neural activity and the vasculature. The neurovascular coupling in the retina is altered in diabetes [223].

Visual stimulation is a powerful modulator of retinal and optic nerve blood flow [224]. Since the blood vessels do not directly respond to light, light-induced changes in blood flow or oxygen delivery must be initiated by light-induced changes in neural activity of the retina. An increase of neural activity leads to retinal arterial and venous dilation [225]. Flicker light stimulation (intermittent flash) has been used to investigate this process, which results altered in diabetic patients without structural microvascular abnormalities in the retina [226–228].

The retina has a high metabolism and O_2_ consumption and, therefore, is particularly susceptible to hypoxia caused by compromised blood flow. The loss of this vascular response could starve the retina of needed oxygen and glucose, putting neurons at risk and contributing to retinal pathology. The improvement of functional hyperaemia could be a useful therapeutic target. In this regard, it has been demonstrated that blocking inducible nitric oxide synthase with systemic administration of aminoguanidine recovers flicker-evoked vasodilation in diabetic retinas [229].

Among the mediators of the hemodynamic impairment of the neurovascular coupling that occurs in diabetic patients, endothelin seems to play a significant role. It should be noted that endothelin 1 (ET-1), a potent vasoconstrictor, is overexpressed in endothelial cells in the setting of DR and enhances glutamate-induced neurotoxicity in retinal neural cells [230, 231]. In addition, it is worth mentioning that, through ETB receptors, ET-1 contributes to retinal ganglion cell loss in rat models of glaucoma and optic nerve injury [231, 232]. Moreover, progressive retinal neurodegeneration has been found in transgenic mice with an overexpression of ET-1 in vascular endothelial cells [233]. Finally, it has been recently demonstrated that the blockade of ETA receptors, apart from ameliorating retinal vascular pathology by reducing pericyte loss and acellular capillaries, prevents thinning in both the optic nerve and retinal periphery in db/db mice [234]. Therefore, ET-1 seems to be a key player involved in the cross-talk between neurodegeneration and vascular abnormalities in the setting of DR. For this reason, the inhibition of ET-1 could lead not only to an improvement in microvascular hemodynamics in the retina, but also to an amelioration of the retinal neurodegeneration associated with diabetes. However, clinical trials are needed to confirm these experimental findings.

#### 3.3.2. Blocking Glutamate Signaling Pathway

Since the extracellular accumulation of glutamate plays a key role in retinal neuron death, treatments addressed at reducing glutamate levels seem appropriate. In this regard, substances targeting the glutamate receptors, such as memantine, have shown beneficial effects on neurodegeneration and vascular abnormalities in streptozotocin-induced diabetic rats [235]. Memantine has failed in demonstrating neuroprotection in phase III clinical trials for glaucoma [236]. However, since the pathophysiology of retinal neurodegeneration is different in diabetes and glaucoma, specific studies examining whether or not memantine may exert a neuroprotective effect in the diabetic retina are needed.

#### 3.3.3. Cell-Based Therapies

Several potential therapies using endothelial precursor cells have been explored. However, the usefulness and safety of these approaches for DR remain to be determined. Regarding retinal development and transplantation strategies, the last decade has seen an enormous progress in the research for photoreceptor replacement and regeneration [237]. Restoration of visual function by cell replacement seems to be a real possibility for neurodegenerative diseases such as retinitis pigmentosa.

## 4. New Perspectives

The advances in both retinal imaging and functional assessment will allow detecting early changes and will accelerate drug discovery and delivery strategies to improve visual prognosis in diabetic patients. In addition, this new approach will encourage the implementation of personalized treatments. In this regard, OCT angiography and confocal adaptive optics scanning ophthalmoscope (AOSLO) are two new technologies that will play a key role in the near future for the diagnosis and monitoring of treatments in early stages of DR. OCT angiography proved to be capable of showing 10 micron-thick blood vessels, revealing the areas of vascular nonperfusion and identifying microexudates that were not otherwise visible on clinical examination and fundus photography. Moreover, OCT angiography allows appreciation of spatial relationships of fundus vessels and makes it possible to separately visualize the large retinal vessels as well as the superficial and the deep capillary plexus [238]. Adaptive optics imaging can be expected to add new aspects to the knowledge of DR because photographic resolution has improved by reducing the influence of optical aberrations on retinal imaging [239]. The confocal AOSLO allows seeing details of the microvasculature, photoreceptors, and hemorheologic parameters and, therefore, is a promising noninvasive and direct method for examining physiopathological events that are taking place in the retina of diabetic patients and may be used for evaluating the effects of clinical interventions [239, 240]. In this regard, clinical trials aimed at examining the effect of controlling the risk factors and new treatments on the diameter of retinal vessels and blood flow would be of particular interest.

From the therapeutic point of view, as previously mentioned, when the early stages of DR are the therapeutic target it would be inconceivable to recommend an aggressive treatment such as intravitreal injections. There is experimental evidence that topical administration is effective for the treatment of early stages of DR [157, 194, 202]. Therefore, the topical route of drug delivery for the treatment of DR could be envisaged as a revolutionary treatment, particularly at the early stages of the disease. However, less than 5% of topically applied dose reaches the deeper ocular tissues [241]. To overcome the ocular drug delivery barriers and improve retinal bioavailability, conventional and novel drug delivery systems are being developed.

In conclusion, the central role of neurodegeneration in the pathogenesis of DR is a solid basis for proposing neuroprotection as an effective strategy for preventing or arresting DR. However, clinical trials to determine not only the effectiveness and safety, but also the compliance of a noninvasive route of drug delivery, as well as a standardization of the methods for monitoring neurodegeneration, are needed. In addition, neuroprotection in the setting of DR should be contemplated as an early treatment; therefore, this exciting experimental approach seems to be still far from acceptance in clinical practice.
